# Challenges in Implementing the Integrated Community-Based Outpatient Therapeutic Program for Severely Malnourished Children in Rural Southern Ethiopia

**DOI:** 10.3390/nu8050251

**Published:** 2016-04-27

**Authors:** Elazar Tadesse, Eva-Charlotte Ekström, Yemane Berhane

**Affiliations:** 1 Department of Women‘s and Children’s Health, International Maternal and Child Health, Uppsala University, Uppsala SE-75185, Sweden; Lotta.Ekstrom@kbh.uu.se; 2 Addis Continental Institute of Public Health, P.O. Box 26751/1000, Addis Ababa, Ethiopia; yemaneberhane@gmail.com

**Keywords:** severe acute malnutrition, ready to use therapeutic foods, outpatient therapeutic program, integrated, Ethiopia

## Abstract

Currently, treatment of uncomplicated severe acute malnutrition is managed in the integrated Community based Outpatient Therapeutic Program (C-OTP) using ready-to-use therapeutic foods (RUTFs). The aim of this study was to determine challenges in implementing the critical steps in C-OTP and caregivers’ perceptions of service provision in southern Ethiopia. A total of 1048 caregivers of children admitted to the OTP and 175 Health Extension Workers (HEWs) from 94 selected health posts were included in the study. Program admission, follow-up and exit information was collected from caregivers during home visits. HEWs were interviewed at their respective health posts. Only 46.6% (481/1032) were given the recommended amount of RUTF and 19.3% (196/1015) were given antibiotics on admission. During C-OTP participation 34.9% (316/905) had uninterrupted provision of the recommended amount of RUTF. Of the children who left the program, 220/554 (39.7%) exited the program in line with the national recommendation. Caregivers (42.9% (394/918) and HEWs (37.1%, 62/167) perceive that RUTFs were being sold as a commodity. Inadequate provision and unintended usage of RUTFs, lack of antibiotics and inappropriate exit from the program were major constraints. For successful saving of lives, adequate resources must be allocated, and providers must be trained regularly, and supervised properly.

## 1. Introduction

Globally, 19 million children under five are suffering from Severe Acute Malnutrition (SAM) [1]. Three out of four children diagnosed with SAM are uncomplicated, and the majority have a good appetite for ready-to-use therapeutic foods (RUTF) and do not have co-morbidities [2,3,4]. The World Health Organization (WHO) recommends the Community-based Outpatient Therapeutic Program (C-OTP) as a standard treatment protocol for the management of uncomplicated SAM at the community level [4,5]. Based on achievements from small-scale programs, C-OTP has been scaled up and integrated into the lowest level government health systems in several developing countries [6,7,8,9] where children are diagnosed, classified and treated for SAM by Community Health Workers (CHWs). CHWs have limited training and have many other responsibilities; thus, one would expect SAM management is likely to differ from small-scale programs which are implemented by non-governmental organizations [3,5].

Once children with uncomplicated SAM are admitted, caregivers visit the C-OTP for check-ups of the nutritional status of their children and the refilling of RUTFs. On average, children stay admitted for 6 to 8 weeks and should be discharged when they achieve program recovery criteria (a gain of 15% admission weight for non-edematous SAM and resolution of edema for those with edema on admission). Recently, the WHO changed the recommendation for discharge from 15% admission weight gain to Mid-Upper Arm Circumference (MUAC) ≥125 mm however, C-OTPs in Ethiopia still use the previous recommendation [3,4]. Children who fail to achieve recovery criteria within the recommended maximum stay in C-OTPs should be transferred to inpatient care for intensive management. The recommended maximum stay in C-OTPs is 8 weeks in the Ethiopian national guideline for SAM management and 6 weeks in the Sphere standard. The Sphere standard has been developed for use in humanitarian response; however, currently it is applied to integrated C-OTP despite ongoing discussions on its use [10,11]. Caregivers who have missed scheduled health post visits and/or whose children fail to improve nutritionally should be visited by the CHWs. The purpose of home visits by the CHWs is to support caregivers by providing child feeding counselling and to supervise the usage of RUTF [12,13]. However, this might not be feasible as CHWs are responsible for providing various preventive and curative health services of which C-OTP is one [14]. In addition, caregivers of children admitted to C-OTP must take on a major share of the SAM management. They are expected to have their children admitted to the program, give medication and feed RUTF at home and bring them for scheduled visits to the health posts [2,4].

As part of the routine health service, integrated C-OTPS operate mostly in a non-emergency context. A variation in recovery rate from the program has been reported since the integration of C-OTP in Ethiopia. Reports from C-OTP integrated to government-run health centers in south-west Ethiopia showed a recovery rate of 45% and the average weight gain was 5–6 g/kg/day which is far below what is ideally expected with from appropriate RUTF consumption [15]. In Ethiopia, prior to scaling up and integration, C-OTPs achieved the Sphere standard of having a high recovery rate, up to 80%, and a low defaulter rate (<5%). However, in the early years of integration, the recovery rate failed to reach 61% and the defaulter rate increased to up to 30% [16]. Inadequate decentralization was one of the reason listed for the deterioration in the program’s performance; thus, the Ministry of Health with technical support from UNICEF continued the scaling-up and integration of the C-OTPs beyond the health centers to include the health posts [6,16]. Recently, a register-based study in Northern Ethiopia found that in scaled-up and integrated C-OTP the recovery rate remained unacceptably low (61.8%) [17]. A similar finding of low recovery (37.8%) was reported from the C-OTPS we included in our study [18]. Examining service delivery at integrated C-OTP has the potential to identify constraints for achieving acceptable program performance. Moreover, since caregivers of admitted children play major role in rehabilitation of SAM in the integrated C-OTPs, assessing their perception of service delivery by the CHWs important. Thus, the aim of this study was to use independently collected data to identify challenges in the implementation of critical steps in C-OTP and caregivers’ perceptions of service provision and RUTF usage in southern Ethiopia.

## 2. Materials and Methods 

### 2.1. Study Design and Setting

This cohort study was part of a larger research project evaluating effectiveness of community-based management of an acute malnutrition program in southern Ethiopia. Children admitted to C-OTPs were identified through a weekly visit to all the health posts in the selected districts. Caregivers of children aged from 6 to 59 months, from whom the research team collected anthropometric data within seven days of admission, and their caregivers were eligible to participate in the study. Four home visits were carried out; the first visit was carried out within one week of admission, and follow-up visits took place at 4, 8 and 14 weeks after admission. Enrolment and data collection are carried out from July to December 2011.

Mostly rural, the residents of the four districts we included in this study engage in crop-livestock mixed farming both for household consumption and marketing. However, recurrent drought and subsequent crop failure in the area has resulted in cyclical nutritional emergencies that have resulted in the districts being recipients of food aid for decades [19,20]. Since 2004, C-OTP has been scaled up and integrated into the government health system in Ethiopia. Guidelines for providing services at community level by Health Extension Workers (HEWs) has been in place since 2007. The HEWs were trained on SAM management and simplified guidelines in local language were prepared which is a key element in the utilization of RUTF innovation [6,21]. HEWs are women CHWs, who have been trained for 10 months through the national Health Extension Program, and who provide basic curative and preventive health services to rural communities for which they are given a salary [3].

### 2.2. Participants and Data Collection

**Figure 1:**
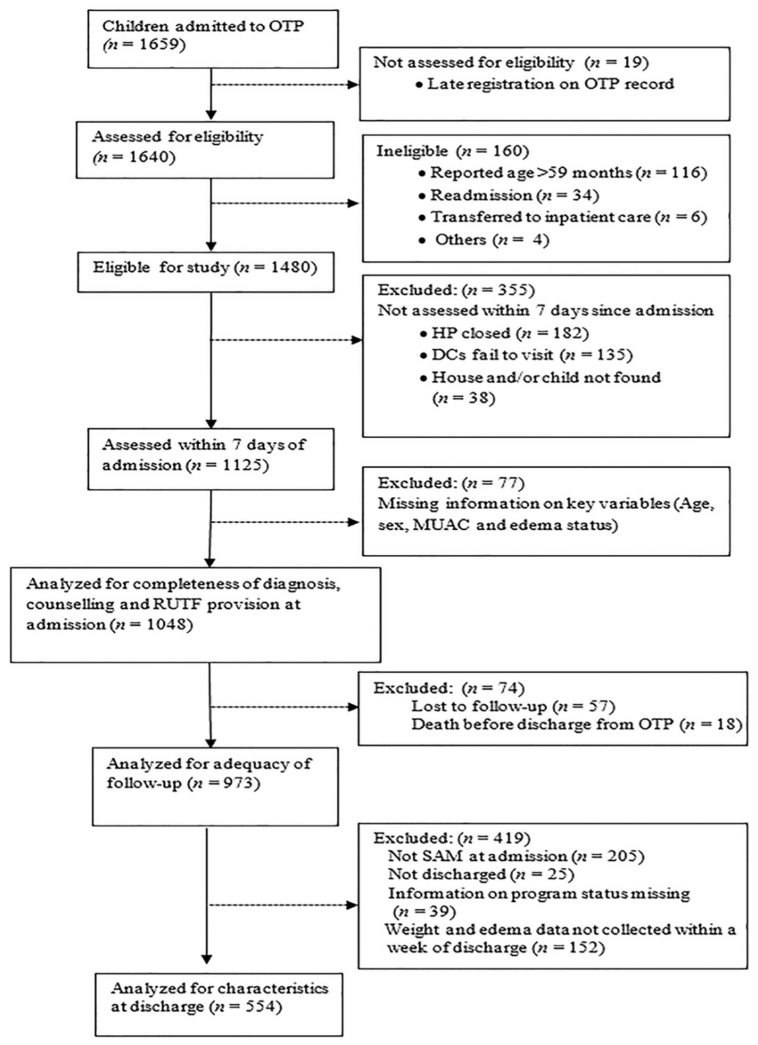
Flow of children admitted to outpatient therapeutic program (OTP). (HP = health post, DC = data collector).

The study participants were caregivers of children admitted to C-OTP and HEWs who provide the service at the 94 health posts. A total of 1048 children admitted to C-OTP and their caregivers were included in this study. Of these, 57 caregivers and their children were lost to follow-up and 18 children died before discharge from the C-OTP (Figure 1). Caregivers were interviewed using a structured questionnaire to collect information on socio-demographic and household characteristics and perceptions of the service provided by the HEWs. All 175 HEWs working in the selected health posts were included in the study. HEWs were interviewed using a structured interview questionnaire regarding their experience of SAM management. Data collectors were 23 women nurses who were not involved in C-OTP implementation and who were not employed by any government health system. They were trained in anthropometric measurement techniques and went through standardization sessions to ensure the accuracy and precision of the information gathered by the measurements according to established guidelines [3]. Responses from HEWs were triangulated with those of the caregivers to add to the validity of our results.

### 2.3. Outcome Measures and Statistical Analysis

The outcome measures in this study were both program- and caregivers-related. The program-related outcomes include the critical steps in SAM management as outlined in the national and WHO guidelines: (1) application of diagnostic criteria for identifying and classifying SAM; (2) provision of recommended amount of RUTF and antibiotics; (3) follow-up during children’s stay in the program; (4) length of C-OTP stay; and (5) application of C-OTP exit procedures. The SAM diagnostic elements were: measuring MUAC; assessing the presence of edema; co-morbidities and carrying out appetite test for RUTF. The former two measurements are to identify SAM while the latter two are for classifying SAM as being either complicated or not [3,22]. Children who were assessed for all the four diagnostic elements were considered as being diagnosed appropriately. RUTFs provision was measured against the recommended amount on admission, and at the 4th and 8th week follow-ups [23]. Key elements of follow-up during the C-OTP stay were measured by performing a check-up of nutritional status (measuring weight and assessing edema status) at the 4th week of admission and length of admission to the program in weeks. The length of C-OTP admission was considered appropriate if it was within the national recommended length of 8 weeks. Follow-up was considered to be appropriate when nutritional status was assessed and the stay in the C-OTP was ≤8 weeks. Unlike the above program-related outcomes, the application of C-OTP exit procedures were measured only for children who had SAM on admission and an anthropometric assessment was done within 7 days of discharge (*n* = 554). Exit from C-OTP was considered appropriate when discharged children achieved the program recovery criteria (a gain of 15% of admission weight for children without edema on admission and resolution of edema for those with edema on admission) while those who failed to achieve the recovery criteria were transferred to inpatient care.

Caregiver-related outcomes were: (1) perception of RUTF usage; (2) experience of attending scheduled health post visits and feeding RUTF to their children; and (3) perception of the service provided by the HEWs. These outcomes were assessed using responses recorded on a four-scale rating (never, few, often and always). The responses were later dichotomized in to “never/few” and “often/always” during analysis.

Data were entered into Epi-Info (Version 6.0) [24], cleaned and exported to SPSS for Windows (Version 20.0), [25] for analysis. Percentage was used for categorical variables and differences in proportion were compared using Chi squared test. Mean ± standard deviation (SD) and median (25th; 75th percentile) were used for continuous variables during analysis and for presentation of the findings.

### 2.4. Ethical Considerations

The Ethical Review Board at Addis Continental Institute of Public Health, Addis Ababa, and the regional ethical review board in Uppsala, Sweden approved the research protocol. A letter of support was obtained from the Regional Health Bureau of South Ethiopia and district health bureaus of the four districts. The purpose and procedures of data collection, confidentiality and voluntary participation was explained to caregivers of children admitted to OTP and verbal consent was obtained from all caregivers as most of the caregivers of admitted children were not able to read and write. All interviews and anthropometric measurements were conducted in privacy during home visits. In order to protect the study participants’ right to confidentiality, the exact location of the study setting is concealed.

## 3. Results

Most of the caregivers (88.6%, 903/1019) of children admitted to OTP were biological mothers and on average, were 30.6 years old. The majority of the children were below 24 months of age (62.3%) and the female-to-male ratio was 1.3. Among children included in the study, 7.3% (77/1048) had edema, 71.5% (749/1048) were severely wasted and the remaining 21.2% (222/1048) were not severely wasted. HEWs were on average 24.7 years old and married (69.9%). The majority, (73.4%) were permanent residents of their work area and almost half of the HEWs (51.8%) lived within <30 min walking distance from the health post where they worked.

### 3.1. SAM Diagnosis and Provision of Treatment

**Figure 2:**
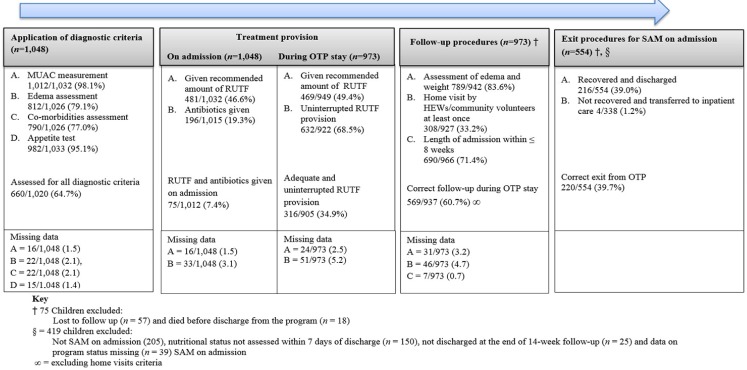
The critical steps in management of Severe Acute Malnutrition (SAM) in a scaled up and integrated OTP.

Figure 2 shows the application of SAM diagnostic criteria and treatment provision in the C-OTP we studied. Almost all children (98.1%, 1012/1032) had their MUAC measured and were given RUTF for an appetite test (95.4%, 982/1033). All SAM diagnostic criteria were assessed for 64.7% (660/1020) of the children. On the other hand, only 46.6% (481/1032) of the children were given the recommended amount of RUTF, and 19.3% (196/1015) of the children were given antibiotics on admission. During follow-up, almost two-third (68.5%, 632/922) had received RUTF provision in all scheduled health post visits however, only 49.9% (469/922) were given the recommended amount thus, only 34.9% (316/905) had adequate and uninterrupted provision of RUTF (Figure 2). The HEWs also reported lack of RUTF as the biggest problem (32.9%, 55/167) they face in the OTP and 62.9% (100/159) reported that they never had antibiotics available at their health posts for children admitted to OTP (Table 1). The proportion of children who achieved program recovery criteria was significantly higher for those children who received antibiotics compared to those treated with RUTFs alone (47.4%, 46/97 *vs.* 52.6, 51/97; *p* = 0.002). The adjusted hazard ratio to achieve program recovery criteria of children who took antibiotics was 1.60 (1.60, 95%-CI: 1.13, 1.16).

### 3.2. Length of Admission, Follow-Up of Nutritional Status and Exit from C-OTP

The majority of the children (71.4%, 690/966) had a length of admission to C-OTP within the national limit of maximum stay in C-OTP which is ≤8 weeks, while 44.0% (425/966) had a length of admission within the Sphere standard limit of maximum stay which is 6 weeks. Almost all children (98.1%, 934/952) had their weight measured and 83.6% (789/942) had their edema status checked during follow-up visits. However, only 33.2% of the caregivers and their children had a home visit either by an HEW or community volunteer. A total of 554 children who had SAM on admission and for whom anthropometric measurements were done within one week of discharge from the program (Figure 1) were included for analysis of appropriateness of exit from C-OTP. From the 554 children who exited the program, 216/554 (39.0%) achieved program recovery criteria (gain of 15% of admission weight for children without edema and resolution of edema for those with edema on admission) and of the 338 who did not achieve the recovery criteria only 4 were transferred to inpatient care. Thus, only 39.8 (220/553) left the program as per the exit options recommended by the national guideline (discharge if program recovery criteria achieved and referral to inpatient care if not).

### 3.3. Perceptions of RUTF Usage and OTP Service Provision

Most of the caregivers (98.1%, 941/959) and 78.2% (129/165) of the HEWs mentioned that getting the malnourished children eat RUTF was not a problem. However, both the caregivers (42.9%, 394/918) and HEWs (37.1%, 62/167) reported that RUTFs were sold as a commodity and 26.4% (245/941) of the caregivers reported that RUTFs were available in shops in their areas. Moreover, 31.6% (300/949) of caregivers mentioned that avoiding sharing RUTF with other children in their household was a problem, even though they were recommended by the HEWs to feed it to only the children admitted to the program (Table 2).

Most caregivers (94.1%, 883/939) perceived that attending the scheduled health post visits was not a problem. Almost two-third of the caregivers (70.4%, 670/952) have a weekly schedule of visiting health posts and all HEWs who were interviewed reported that it was not a problem to get mothers come to the health posts as per their schedule. Almost all the caregivers reported that HEWs adequately listened (93.9%, 905/964) and understood their situation (94.2%, 909/965) and had sufficient time with them (94.6%, 913/965) (Table 3).

## 4. Discussion

In the studied C-OTPs both service provision at the health posts and caregivers’ management at their homes were constrained. Only 1 in 4 children admitted to the program had adequate and uninterrupted RUTF provision and only 1 in 35 children who failed to achieve program recovery criteria at exit from the program were transferred to inpatient care. Only 1 in 3 had a home visit either by an HEW or community volunteer. However, caregivers perceived that HEWs adequately listened, understood their situation and had sufficient time with them during the scheduled health post visits. Moreover, RUTFs were perceived to be sold and shared to meet other purposes than the program’s intention.

### 4.1. Service Provision-Related Constraints

The major constraints in service provision were the inability to provide the recommended amount of RUTF and the inappropriate exit of admitted children from C-OTPs. Less than half of the children (46.6%) on admission and only 49.9% during follow-up received the recommended amount of RUTFs. Health posts should get RUTFs from their respective district health offices monthly [26], however, during data collection we observed a severe shortage of transportation means to get RUTFs to the health posts although the district health office had it in stock. Both caregivers of admitted children and HEWs mentioned that the supply of medications and other program supplies to this area is inadequate and interrupted. The same was reported about the program in Bangladesh, where the supplies are provided through the health system rather than NGOs [27]. Moreover, reviews of various research findings from several community-based health services showed that shortage of resources was the main reason for the failure of the CHWs to fulfil their work responsibilities [28,29].

The second challenge in the implementation of a scaled-up and integrated C-OTP is the exit of severely malnourished children from C-OTP who have neither achieved program recovery criteria nor have been transferred to inpatient care. Failure to achieve program recovery criteria can result from inadequate consumption of RUTF by the admitted children because of inadequate provision both at the health post and at home. Moreover, studies from the same area reported that HEWs were seen discharging children on the basis of the maximum stay (8 weeks) in the C-OTPs [13]. Previous studies also showed that discharge from C-OTP is perceived as temporary by caregivers and they expect their children to be back in C-OTP in 2–3 weeks [13]. Limited availability of inpatient care and a weak referral network between health posts and higher health institutions are barriers to the transfer of children who have failed to achieve the recommended program criteria upon their exit from the program [12].

Thirdly, only 19.3% of admitted children were given antibiotics and the majority (62.9%) of the HEWs reported that they never had antibiotics in their OTPs. The WHO as well as the Ethiopian national guideline recommends the use of broad spectrum antibiotics for all children admitted to C-OTP with a diagnosis of uncomplicated SAM [3,5]. In Ethiopia, HEWs are allowed to provide antibiotics to children admitted to the program [3,26]. However, the C-OTPs we studied suffered a severe shortage of antibiotics. Previously, researchers were skeptical about the use of antibiotics to manage of children with uncomplicated SAM and highlighted the risk of antibiotic resistance that can result from such blanket use [30,31]. In contrast, a recent study by Trahan *et al.* that had large a sample size showed significant improvement in the children’s recovery and a reduction in the mortality rate with the use of antibiotic [32]. The 2013 updated WHO guideline for the management of SAM recommends the use of antibiotics for the management of SAM in C-OTPs [4]. In this study, the proportion of children achieving the program recovery criteria was significantly higher for those who received antibiotics than those who were treated with RUTF alone. Amoxicillin is the most commonly used antibiotic in the management of uncomplicated SAM in Ethiopia [3].

### 4.2. Constraints Related to Home-Based Management

RUTFs that were given for consumption by children admitted to C-OTPs were perceived to be used as a commodity and RUTFs were available in shops in the study area. Avoiding the sharing of RUTF with other children in the household was difficult. Our study was conducted among a population that has been facing household food insecurity and extreme poverty. In such a context available resources, including RUTF and other nutritional and food interventions, are used by the community for collective preservation of their precarious livelihood [13,33,34]. Moreover, the very characteristics of RUTFs that makes it suitable for the program also make it easily bartered for a reasonable amount of money that enables extremely poor households to obtain cash for purchasing basic household commodities and cheap food that can be shared by all family members [13]. Thus, unintended use of RUTF has the potential to undermine the effectiveness of a C-OTP which is already constrained by inadequate and/or interrupted RUTF provision. The SAM management protocol in Ethiopia recommends home visits by HEWs and/or community volunteers to support caregivers and address conditions that delay/hinder the recovery of SAM children [3,5]. In our study, almost two-third of caregivers never had any such home visits during their children’s stay in C-OTP. The home visit element was found to be the weakest activity of the C-OTP service [12].

A strength of this study is that data on the elements of service delivery at admission, during stay and exit from C-OTP were collected in a timely manner (*i.e.*, within the first week of admission and while the children were still admitted). The study was completed by an independent team that was not involved in any aspect of the program or any other health and nutrition interventions in the study area. A limitation of this study is that diagnostic criteria were used but were not observed for correctness of techniques used for assessment thus, the proportion of children with proper diagnosis might not reflect appropriateness of the diagnostic criteria assessed.

## 5. Conclusions

The implementation of an integrated C-OTP was constrained, both from the program- and home-based management sides. Inadequate provision of RUTF by the service providers and unintended usage of RUTFs by caregivers of admitted children have the potential to delay and/or hinder the nutritional recovery of SAM children and thus lead to chronic nutritional deprivation of the children. Efficient provision and proper use of RUTFs is critical for reducing childhood severe acute malnutrition. Thus, availability of RUTFs should be ensured through application of appropriate supply chain and monitoring policy. In addition, nutrition interventions that address household food insecurity has the potential to reducing sharing and selling of RUTFs, thus improve effectiveness of SAM management at integrated C-OTPs. This might need a policy platform that brings together different stakeholders such as local government, non-government organization and international organizations. The other challenges in integrated C-OTP implementation were lack of antibiotics, lack of transfer of children who fail to achieve program recovery criteria to inpatient care, and inadequate home visits by the HEWS were major challenges. To ensure the success of C-OTPs in saving lives, adequate antibiotics need be made available, providers must be trained regularly, and regular supportive supervision must be in place.

## Figures and Tables

**Table 1:** Health extension workers’ perceptions of resource availability at their health posts (*n* = 175).

Perceptions of Resource Availability	Missing Data *n (%)*	*n/n*	%
Lack of RUTF in the health post is a problem	8 (4.5)		
Never/small problem		112/167	67.1
Common problem		55/167	32.9
Antibiotic not available in the health post (the longest time)	16 (9.1)		
1 week–1 month		12/159	7.5
>1 month		47/159	29.6
Never had antibiotic		100/159	62.9
HEW often feel overwhelmed because of all they work	9 (5.1)		
Strongly disagree/disagree		19/166	11.4
Neither agree or disagree		0/166	0.0
Strongly agree/agree		147/166	88.6
HEW feels that the time they have is sufficient to complete all expected the activities	8 (4.5)		
Strongly disagree/disagree		109/167	65.3
Neither agree or disagree		1/167	0.6
Strongly agree/agree		57/167	34.1

**Table 2:** Perceptions of Ready to Use Therapeutic Food (RUTF) usage among caregivers of children admitted to OTP and HEWs (*n* = 973 caregivers, *n* = 175 HEWs).

Perceptions of Usage of RUTF	Caregivers		HEWs	
	Missing Data *n* (%)	*n*/*n* (%)	Missing Data *n* (%)	*n*/*n* (%)
It is a problem to make malnourished children eat RUTF	14 (1.4)		10 (5.7)	
Never difficult		941/959 (98.1)		129/165 (78.2)
Sometimes difficult		18/959 (1.9)		36/165 (21.8)
Caregivers selling of RUTF is a problem	55 (5.6)		8 (4.6)	
It is not a/small problem		424/918 (57.1)		105/167 (62.9)
It is a/large problem		394/918 (42.9)		62/167 (37.1)
Is it difficult to avoid sharing RUTF with other non-SAM children in the household	24 (2.5)			
Not difficult		635/949 (66.9)		NA
Difficult		300/949 (31.6)		NA
No other children in the HH		14/949 (1.5)		NA
RUTFs available in shops	32 (3.2)			
Yes		248/941 (26.4)		NA
Not sure		642/941 (68.2)		
No		51/941 (5.4)		

NA = Not assessed.

**Table 3:** Caregivers’ views of community-based outpatient therapeutic program service provision (*n* = 973).

Item	Missing Data *n (%)*	*n/n (%)*
How difficult is it for you to have this schedule for collection of RUTF (*n* = 939)	34 (3.5)	
Not difficult		883/939 (94.0)
A little/very difficult		56/939 (6.0)
How often should you visit the health post to collect RUTF if you have a child with SAM (*n* = 952)	21 (2.2)	
More than once in a week		282/952 (29.6)
Once in a week		670/952 (70.4)
To what extent do you think distance to the health post is a problem to adhere to follow up visits for SAM children (*n* = 946)	6 (0.6)	
Not at all/small problem		782/967 (80.9)
Big problem		185/967 (19.1)
Do caregivers think HEWs listen to their problems? (*n* = 964)	9 (0.9)	
Yes		905/964 (93.9)
No		59/964 (6.1)
Do caregivers think the HEW spent sufficient time with them and their children when visiting the health post for SAM? (*n* = 965)	8 (0.8)	
Yes		909/965 (94.2)
No		56/965 (5.8)
Do caregivers think the HEWs were able to understand their problems/challenges related to managing their SAM child management? (*n* = 965)	8 (0.8)	
Yes		913/965 (94.6)
No		52/965 (5.4)

## Abbreviations

The following abbreviations are used in this manuscript:

CHW	Community Health Workers
C-OTP	Community-based Outpatient Therapeutic Program
HEW	Health Extension Workers
MUAC	Mid-Upper Arm Circumference
SAM	Severe Acute Malnutrition
WHO	World Health Organization
